# Domestic Violence Police Reporting and Resources During the 2020 COVID-19 Stay-at-Home Order in Chicago, Illinois

**DOI:** 10.1001/jamanetworkopen.2021.22260

**Published:** 2021-09-02

**Authors:** Louisa Baidoo, Tanya L. Zakrison, Gillian Feldmeth, Stacy Tessler Lindau, Elizabeth L. Tung

**Affiliations:** 1 University of Chicago Pritzker School of Medicine, Chicago, Illinois; 2 Section of Trauma and Acute Care Surgery, Department of Surgery, University of Chicago, Chicago, Illinois; 3 NowPow, CareIT Health, LLC; 4 Department of Obstetrics & Gynecology, University of Chicago, Chicago, Illinois; 5 Section of General Internal Medicine, Department of Medicine, University of Chicago, Chicago, Illinois; 6 Center for Health and the Social Sciences, University of Chicago, Chicago, Illinois

## Abstract

**Importance:**

Domestic violence (DV) has become a growing public health concern during the COVID-19 pandemic because individuals may be sheltering in place with abusers and facing mounting economic and health-related stresses.

**Objective:**

To analyze associations of the 2020 COVID-19 stay-at-home (SH) order with DV police reporting and resource availability, including differences by community area racial/ethnic composition.

**Design, Setting, and Participants:**

This longitudinal cohort study assessed DV police reports (January-June 2020) obtained from the Chicago, Illinois, Police Department and DV resource availability (March and August 2020) obtained from the NowPow community resource database, both for 77 community areas in Chicago. Data were analyzed July through December 2020.

**Exposures:**

The COVID-19 SH order effective March 21, 2020.

**Main Outcomes and Measures:**

Monthly rates of DV police reports and DV resource availability per 100 000 persons.

**Results:**

Of 77 community areas in Chicago, 28 (36.4%) were majority Black, 19 (24.7%) majority Hispanic/Latinx, 18 (23.4%) majority White, and 12 (15.6%) a different or no majority race/ethnicity, representing an estimated population of 2 718 555 individuals. For each community area, the SH order was associated with a decrease in the rate of DV police reports by 21.8 (95% CI, −30.48 to −13.07) crimes per 100 000 persons per month relative to the same months in 2019. Compared with White majority community areas, Black majority areas had a decrease in the rate of DV police reports by 40.8 (95% CI, −62.93 to −18.75) crimes per 100 000 persons per month relative to the same months in 2019. The SH order was also associated with a decrease in DV resource availability at a rate of 5.1 (95% CI, −7.55 to −2.67) resources per 100 000 persons, with the largest decreases for mental health (−4.3 [95% CI, −5.97 to −2.66] resources per 100 000 persons) and personal safety (−2.4 [95% CI, −4.40 to −0.41] resources per 100 000 persons). The Black majority south side of Chicago had a larger decrease in resource availability (−6.7 [95% CI, −12.92 to −0.46] resources per 100 000 persons) than the White majority north side.

**Conclusions and Relevance:**

In this longitudinal cohort study, the rate of DV police reports decreased after the SH order was implemented in Chicago. This decrease was largely observed in Black majority communities, whereas there was no significant change in White majority communities. These findings may reflect decreased DV incidence but may also reflect an exacerbation of underreporting. In addition, DV resource availability decreased disproportionately on the predominantly Black south side of Chicago.

## Key Points

**Question:**

Are there associations between the COVID-19 stay-at-home order issued March 2020 in Chicago, Illinois, and the rate of domestic violence (DV) police reporting and resource availability?

**Findings:**

In this cohort study of 77 Chicago community areas, the stay-at-home order was associated with a decrease in the rate of DV police reports by 21.8 crimes per 100 000 persons per month relative to the same months in 2019, a finding observed largely in Black communities, with no significant change in White communities. Resource availability decreased by 5.1 resources per 100 000 persons.

**Meaning:**

The decreased rate of DV police reports during the stay-at-home order, especially in Black majority communities, may be due to decreased DV incidence or exacerbated underreporting; resource availability also decreased on the predominantly Black south side of Chicago.

## Teaser

This cohort study assesses whether a COVID-19 stay-at-home order was associated with changes in police report rates or domestic violence resource availability that differed by racial/ethnic majority compositions in 77 community areas of Chicago, Illinois.

## Introduction

Domestic violence (DV) has been a public health concern during the COVID-19 pandemic.^1^ Domestic violence includes any physical, sexual, psychological, or other violent behavior perpetrated by a family member, partner, or household resident.^2^ In minimizing COVID-19 spread through social distancing and lockdown measures, officials balanced public health and safety against economic progress and community well-being. Early vaccine inaccessibility and emerging concerns about vaccine-resistant strains^3^ compelled a focus on isolation to prevent transmission. However, because isolation is a key risk factor for DV,^4^ those measures may have inadvertently increased risk of DV. For example, at the start of the pandemic, the DV website traffic for New York City more than doubled,^5^ and the National Domestic Violence Hotline reported an increase in callers disclosing that the pandemic had worsened their circumstances.^6^

Criminologists theorize that DV may be associated with negative coping mechanisms in the context of stress.^7^ Experts have raised concerns about an increase in DV during the pandemic owing to prolonged contact with abusers, financial stress, and overwhelmed emergency and community resources.^8,9,10^ Furthermore, DV rates tend to increase after public crises.^9,10,11^ Although media and several studies^8,11^ have reported on DV during the COVID-19 pandemic, few studies have examined how the pandemic may have changed opportunities to mitigate DV.

The present study primarily aimed to evaluate whether the March 2020 stay-at-home (SH) order in Chicago, Illinois,^12^ was associated with changes in DV police reporting, and how police reporting varied by community area race/ethnicity. We hypothesized that DV police reporting increased, corresponding with a rise in DV incidence during the SH order, as stress and opportunity for domestic conflict increased. However, DV is markedly underreported owing to factors such as lack of legal support, fear of escalation, and stigma.^13,14,15^ Thus, it is also plausible that the SH order exacerbated underreporting, as support to escape violence decreased. Thus, our secondary aim was to evaluate how DV resource availability changed during the pandemic. We hypothesized that DV resource availability decreased as organizations adapted to the SH order.

## Methods

### Setting and Data Sources

This study was conducted in Chicago, Illinois, which is composed of 77 sociologically and historically distinct community areas. Chicago is also divided into north, west, and south sides. The north side is majority White race/ethnicity, with 54.2% White, 27.0% Hispanic/Latinx, and 6.8% Black residents. The south side is majority Black race/ethnicity, with 68.4% Black, 13.1% White, and 11.7% Hispanic/Latinx residents. The west side is racially/ethnically heterogeneous compared with the other 2 sides, with 45.3% Hispanic/Latinx, 32.6% Black, and 17.5% White residents.^16^ The reporting of this study follows the Strengthening the Reporting of Observational Studies in Epidemiology (STROBE) reporting guideline for cohort studies.^17^ This study did not meet the definition of Human Subjects Research by the University of Chicago Institutional Review Board because it included only deidentified and publicly available data.

We analyzed 2019-2020 crime report data from the Chicago Police Department Citizen Law Enforcement Analysis and Reporting system, which collects information on crimes reported to the Chicago Police Department, including location, date, and crime type. This database contains only crimes reported to police. For the present study, domestic crime refers to crimes occurring in residential locations.

Domestic violence resource data were obtained from NowPow, a community resource referral platform that maintains comprehensive directories of information about area resources in several geographies, including Chicago. Through research or direct contact, NowPow assesses resource availability at least semiannually. Domestic violence resource data included name, type, location, and availability during the COVID-19 pandemic.^18,19^ Beginning March 2020, essential resource availabilities were updated at least biweekly. Data used in the present study reflect availabilities as of August 2020.

Community area race/ethnicity data were obtained using 2018 American Community Survey 5-year population estimates.^16^ Community areas were classified as majority Black, Hispanic/Latinx, White, or other race/ethnicity (ie, a different race/ethnicity or no majority). A majority was defined as more than 50% of residents identifying with a particular racial/ethnic group.

### Design

The DV police reporting analysis approximated a longitudinal cohort design by treating community areas as subjects, with repeated observations by month during a 6-month study period. The 2020 COVID-19 SH order went into effect at the end of March and served as the “exposure,” delineating the period before the SH order (January-March) from the period after the SH order (April-June). The same months in 2019 served as controls to account for seasonal crime variation.^20^

The DV resource analysis assessed resource availability at 2 time points: (1) before March 2020 (before the COVID-19 pandemic) and (2) August 2020 (during the COVID-19 pandemic). A control group was unnecessary because changes in availability were directly attributable to the pandemic (ie, services reported a “COVID-19 status”) and were not prone to seasonal variation.

### Measures

We categorized domestic crime reports into 3 types: (1) DV (homicide, manslaughter, assault or battery, sexual assault, robbery, and stalking), (2) property crimes (theft and property damage), and (3) other crimes (eg, weapons violations, drug crimes, and offenses involving children). The primary dependent variable was the rate of DV police reports, measured in crimes per 100 000 persons per month. The rates of domestic property crimes and other crimes were analyzed for comparison. Domestic and nondomestic homicides were also analyzed for comparison because although nonfatal DV is underreported, homicides are fully or almost fully reported to police and may serve as a better indicator of incidence.

The secondary dependent variable was the rate of DV resource availability, measured in resources per 100 000 persons. Resource rates were calculated for each community area. Four types of resources were analyzed: (1) legal assistance (ie, advocacy and court accompaniment services), (2) mental health (ie, counseling services), (3) personal safety (ie, DV shelters, survivor support services, anger management classes, and DV prevention education), and (4) hotlines (ie, crisis, DV, and sexual assault hotlines).

Sociodemographic covariates included median age, median household income, and educational attainment (ie, proportion of residents with a high school diploma) for each community area. We also included variables for vehicle access and group transportation. Vehicle access may provide individuals with the physical mobility to escape danger. Group transportation, which may have posed a health risk during the COVID-19 pandemic, was a measure of the proportion of commuters in a community area who relied on public transit or carpooling.

### Statistical Analysis

For each domestic crime type (violent, property, other, and homicide), mixed-effects linear regression models, including a random intercept for each community area, were used to calculate the change in rates of police reporting (per community area per month) as a function of the interaction between period (before SH order vs after SH order) and year (2019 vs 2020; model 1). Given the high degree of racial/ethnic segregation in Chicago^21^ as well as racial/ethnic variation in both policing^22^ and COVID-19 disease burden,^23^ we also tested for interactions with the racial/ethnic composition of each community area (model 2). Final models included all aforementioned theoretically relevant covariates.

For secondary analyses, we geocoded each DV resource from NowPow’s database and quantified the number of resources per 100 000 persons within community areas. All resources included in the analysis were confirmed to be available at least once in the 6 months prior to the pandemic (before COVID-19). Resources with a COVID-19 status listed as “virtual” or “regular operations” were considered available during the pandemic. Resources with a status listed as “unavailable” or “unable to verify” (ie, the resource was confirmed as available before the COVID-19 pandemic, but the research team was unable to verify status during the COVID-19 pandemic) were considered unavailable. For each resource type (legal assistance, mental health, and personal safety), mixed-effects linear regression models, including a random intercept for each community area, were used to calculate the change in accessible resources (number of operating resources per 100 000 persons) as a function of time period (before the COVID-19 pandemic vs during the COVID-19 pandemic; model 1). Hotline availability was not analyzed because hotlines did not experience changes in operational status and were available to individuals regardless of physical location. Similar to the aforementioned analyses, we tested for interactions between time period and the racial/ethnic composition of each community area (model 2).

A 2-sided value of *P* < .05 was considered statistically significant. All analyses were conducted using Stata IC, version 16 (StataCorp LLC).

## Results

### Overall Trends and Community Characteristics

**Figure 1.**
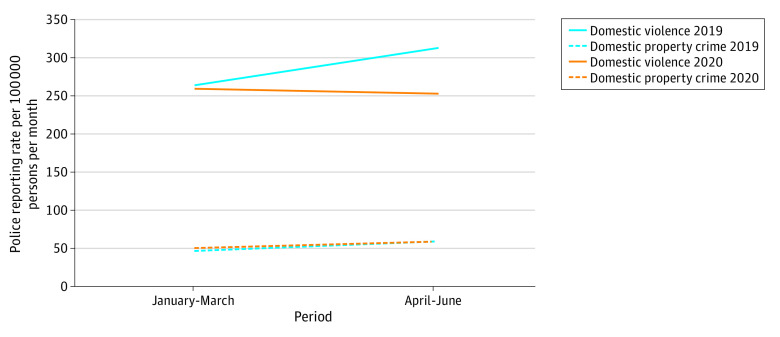
Changes in Domestic Violence and Domestic Property Crime Reporting Rates Before vs After Implementation of the 2020 COVID-19 Stay-at-Home Order in Chicago

On the basis of an estimated population of 2 718 555 individuals (eTable 1 in the Supplement),^16^ the rates of DV police reports were similar in January through March of 2019 (264.0 crimes per 100 000 persons per month) and before SH orders were implemented in January through March of 2020 (259.8 crimes per 100 000 persons per month). However, rates of DV reports differed in April through June 2019 (313.2 crimes per 100 000 persons per month) compared with April through June 2020 (253.0 crimes per 100 000 persons per month) after the SH orders were implemented. By contrast, domestic property crime reports followed similar trends in 2020 compared with 2019 (Figure 1). A month-to-month analysis indicated that DV police reports reached a 5-year low of 70.1 crimes per 100 000 persons in April 2020 immediately after the SH order was implemented (eFigure in the Supplement).

Sociodemographic characteristics for Chicago’s 77 community areas are given in Table 1. One-third (36.4%) of community areas contained majority Black residents, one-quarter (24.7%) contained majority Hispanic/Latinx residents, and another one-quarter (23.4%) contained majority White residents. Fewer (15.6%) community areas had another racial/ethnic majority or no majority. The median household income was $48 966.67 (interquartile range, $33 419.90-$66 309.30), with 6.5% of community areas having a median household income less than $25 000. Few community areas (18.2%) had low household vehicle access (ie, <60% of residents with vehicle access); two-thirds (68.9%) had moderate or high levels of group transportation use (ie, ≥35% residents reporting carpool or public transit use) (Table 1).

**Table 1.** Characteristics of 77 Community Areas in Chicago

Community area characteristic	No. (%)
Race/ethnicity majority	
Non-Hispanic White	18 (23.4)
Non-Hispanic Black	28 (36.4)
Hispanic/Latinx	19 (24.7)
Other/none^a^	12 (15.6)
Median age, y^b^	
20-29	4 (5.2)
30-39	57 (74.0)
40-49	15 (19.5)
50-60	1 (1.3)
Median household income, $	
<25 000	5 (6.5)
25 000-49 999	35 (45.4)
50 000-74 999	22 (28.6)
75 000-99 999	9 (11.7)
≥100 000	6 (7.8)
Educational attainment^c^	
Low	8 (10.4)
Moderate	50 (64.9)
High	19 (24.7)
Household vehicle access^d^	
Low	14 (18.2)
Moderate	29 (37.7)
High	34 (44.2)
Group transportation use^e^	
Low	24 (31.2)
Moderate	41 (53.3)
High	12 (15.6)

^a^ Other includes community areas with a majority of residents identifying as non-Hispanic Asian, Native Hawaiian and other Pacific Islander, American Indian and Alaska Native, “some other race,” or multiracial, as designated by the US Census Bureau American Community Survey.^16^

^b^ Estimates were derived from age group frequencies.

^c^ Percentage of community area population 25 years of age or older with a high school degree or higher: low, 50% to 69%; moderate, 70% to 89%; and high, 90% to 100%.

^d^ Percentage of households with access to at least 1 vehicle: low, 40% to 59%; moderate, 60% to 79%; and high, 80% to 100%.

^e^ Percentage of commuters 16 years of age or older who either carpooled or took public transportation: low, 0% to 34%; moderate, 35% to 44%; and high, 45% to 100%.

### Domestic Violence Police Reporting

Per community area, the rate of DV police reports decreased by 21.8 (95% CI, −30.48 to −13.07) crimes per 100 000 persons per month (*P* < .001) (Table 2) after implementation of the 2020 SH order relative to 2019. The rates of police reports of domestic property crime (−1.0 [95% CI, −4.53 to 2.49] crimes per 100 000 persons per month) and other crime (0.0 [95% CI, −3.37 to 3.31] crimes per 100 000 persons per month) did not change significantly. White majority community areas experienced no significant decrease in the rate of police reports (−4.4 [95% CI, −21.64 to 12.83] crimes per 100 000 persons per month; *P* = .62) (eTable 2 in the Supplement), whereas Black majority community areas experienced a substantial decrease by 45.2 (95% CI, −59.06 to −31.43) crimes per 100 000 persons per month (*P* < .001). The difference between White racial/ethnic community areas and Black racial/ethnic community areas was statistically significant (−40.8 [95% CI, −62.93 to −18.75] crimes per 100 000 persons per month) (*P* < .001) (Table 2).

**Table 2.** Change in Domestic Crime Reporting Rates, Relative to 2019, Associated with Implementation of the 2020 COVID-19 Stay-at-Home Order in Chicago

Crime type and racial/ethnic majority composition	Model 1^a^		Model 2^b^	
	Change in crime reports per 100 000 persons/mo (95% CI)	*P* value	Change in crime reports per 100 000 persons/mo (95% CI)	*P* value
Violent	−21.8 (−30.48 to −13.07)	<.001		
White			1 [Reference]	
Black			−40.8 (−62.93 to −18.75)	<.001
Hispanic/Latinx			−3.8 (−27.89 to 20.20)	.75
Other/none^c^			−10.1 (−37.32 to 17.17)	.47
Property	−1.0 (−4.53 to 2.49)	.57		
White			1 [Reference]	
Black			−0.9 (−10.06 to 8.24)	.85
Hispanic/Latinx			0.7 (−9.30 to 10.61)	.90
Other/none^c^			1.5 (−9.78 to 12.78)	.79
Other^d^	0.0 (−3.37 to 3.31)	.99		
White			1 [Reference]	
Black			4.4 (−4.22 to 13.05)	.32
Hispanic/Latinx			−4.7 (−14.06 to 4.74)	.33
Other/none^c^			−1.1 (−11.79 to 9.51)	.83
Total	−22.8 (−33.16 to −12.49)	<.001		
White			1 [Reference]	
Black			−37.3 (−63.39 to −11.28)	.01
Hispanic/Latinx			−7.8 (−36.22 to 20.52)	.59
Other/none^c^			−9.7 (−41.86 to 22.42)	.55

^a^ Model 1 implemented mixed-effects linear regression models to calculate the change in police reporting rates (per community area per month) as a function of the interaction between period (before vs after SH order) and year (2019 vs 2020), controlling for median age, median household income, educational attainment, vehicle access, and group transportation.

^b^ Model 2 implemented all conditions of model 1 but additionally stratified by each community area racial/ethnic composition.

^c^ Other includes community areas with a majority of residents identifying as non-Hispanic Asian, Native Hawaiian and other Pacific Islander, American Indian and Alaska Native, “some other race,” or multiracial, as designated by the US Census Bureau American Community Survey.^16^

^d^ Other includes, for example, weapons violations, drug crimes, and offenses involving children.

Although the rates of DV police reports decreased after implementation of the SH order, the rates of homicides increased (eTable 3 in the Supplement). The increase in the number of total homicides was larger in 2020 (change, 138) compared with 2019 (change, 82), with a relative difference increase of 0.2 (95% CI, 0.01-0.48) homicides per community area per month (*P* = .04) (eTable 4 in the Supplement). Owing to the low number of domestic homicides during the 6-month study period, there was insufficient power to analyze domestic homicides alone; thus, we analyzed domestic and nondomestic homicides together.

### Domestic Violence Resource Availability

Prior to the pandemic, 552 DV resources were available at a rate of 20.3 resources per 100 000 persons per community area. A vast majority of resources (72.6%) were mental health resources, which operated at a rate of 14.8 resources per 100 000 persons. Of the remaining resources, 18.7% were personal safety (3.8 resources per 100 000 persons), 6.9% were hotlines (1.4 resources per 100 000 persons), and 1.8% were legal assistance (0.4 resources per 100 000 persons; Table 3) resources. Overall, one-fifth (19.5%) of DV resources were considered closed (COVID-19 status unavailable or unable to verify), including a large proportion of anger management classes (31.1%), counseling services (21.0%), and DV prevention educational services (20.0%). However, DV shelters did not experience any closures (eTable 5 in the Supplement).^24^

**Table 3.** Change in Domestic Violence Resource Availability Rates in Chicago Associated With the COVID-19 Pandemic

Resource type and racial/ethnic majority composition	Prior to COVID-19, resources per 100 000 persons	Model 1^a^		Model 2^b^	
		Change in resources per 100 000 persons (95% CI)	*P* value	Change in resources per 100 000 persons (95% CI)	*P* value
All types^c^	20.3	−5.1 (−7.55 to −2.67)	<.001		
White				1 [Reference]	
Black				−5.4 (−11.67 to 0.95)	.10
Hispanic/Latinx				−0.4 (−7.27 to 6.48)	.91
Other/none^d^				−0.9 (−8.66 to 6.92)	.83
Legal assistance	0.4	−0.2 (−0.44 to 0.05)	.13		
White				1 [Reference]	
Black				0.0 (−0.66 to 0.66)	>.99
Hispanic/Latinx				−0.4 (−0.91 to 0.02)	.06
Other/none^d^				-	
Mental health	14.8	−4.3 (−5.97 to −2.66)	<.001		
White				1 [Reference]	
Black				−3.1 (−7.30 to 1.19)	.16
Hispanic/Latinx				−0.5 (−5.32 to 4.39)	.85
Other/none^d^				0.0 (−5.04 to 5.10)	.99
Personal safety	3.8	−2.4 (−4.40 to −0.41)	.02		
White				1 [Reference]	
Black				−4.9 (−10.09 to 0.37)	.07
Hispanic/Latinx				−0.7 (−6.30 to 4.84)	.80
Other/none^d^				−0.7 (−6.76 to 5.29)	.81

^a^ Model 1 implemented mixed-effects linear regression models to calculate the change in resource availability (per community area) as a function of period (before vs during the COVID-19 pandemic), controlling for median age, median household income, educational attainment, vehicle access, and group transportation.

^b^ Model 2 implemented all conditions of model 1 but additionally stratified by each community area racial/ethnic composition.

^c^ Includes legal assistance, mental health, personal safety, and hotline resources.

^d^ Other includes community areas with a majority of residents identifying as non-Hispanic Asian, Native Hawaiian and other Pacific Islander, American Indian and Alaska Native, “some other race,” or multiracial, as designated by the US Census Bureau American Community Survey.^16^

**Figure 2.**
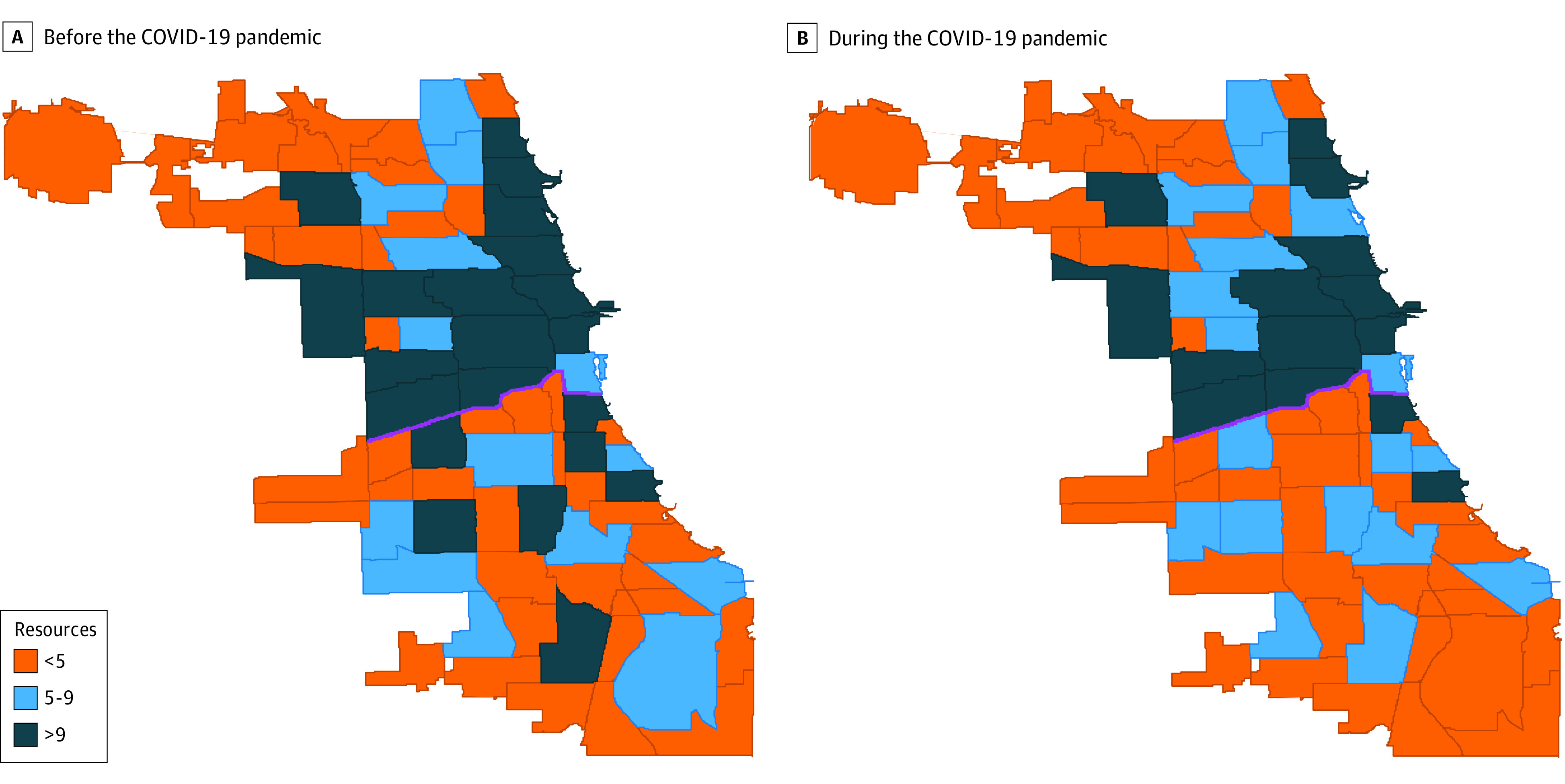
Availability of Domestic Violence Resources in Chicago Before and During the COVID-19 Pandemic by Community Area Change in the number of available domestic violence resources before and during the COVID-19 pandemic. The solid purple line demarcates the North and South Sides of Chicago.

Per community area, DV resource availability decreased by a rate of 5.1 (95% CI, −7.55 to −2.67) resources per 100 000 persons, a 25.1% reduction in total resource availability. Mental health resource availability decreased at a rate of 4.3 (95% CI, −5.97 to −2.66) resources per 100 000 persons (29.1% reduction) and personal safety resource availability decreased at a rate of 2.4 resources (95% CI, −4.40 to −0.41) per 100 000 persons (63.1% reduction). Changes in legal assistance availability were not significant (−0.2 [95% CI, −0.44 to 0.05] resources per 100 000 persons) and hotlines remained available throughout the pandemic (Table 3). Black majority community areas showed decreases in resource availability at a rate of 5.4 (95% CI, −11.67 to 0.95) resources per 100 000 persons relative to White majority community areas, but these differences were not statistically significant (*P* = .10). However, south side community areas, which include predominantly Black residents, showed decreases in resource availability at a rate of 6.7 (95% CI, −12.92 to −0.46) resources per 100 000 persons (*P* = .04) relative to north side community areas (Figure 2; eTable 6 in the Supplement).

## Discussion

In this longitudinal cohort study of 77 community areas in Chicago, the COVID-19 SH order issued March 21, 2020, was associated with a decrease in DV police reports at a rate of 21.8 crimes per 100 000 persons per month, relative to 2019. This decrease was observed despite increased DV hotline traffic and pandemic-related stressors. There are 2 potential explanations for our findings. It is possible that DV incidents decreased, resulting in fewer police reports; alternatively, it is possible that individuals experienced DV at consistent or higher rates but did not report to police. Several of our findings support the latter hypothesis—that COVID-19 exacerbated known DV underreporting and additionally worsened racial disparities in underreporting.

First, homicide rates, which are substantially less likely to be underreported to police, increased during the SH order. It is possible that violent incidents occurred at consistent or higher rates, but at times escalated with decreased uptake of emergency services. In addition, police reports of domestic property crimes in 2020 followed nearly identical patterns compared with 2019. Those crimes often do not involve household members (eg, burglary) and are also less likely to be underreported to police.

Second, Black communities experienced a substantial decrease in DV police reporting while White communities did not. The pandemic coincided with political actions following the deaths of Breonna Taylor, a Black woman killed by police in Louisville, Kentucky, in March 2020, and George Floyd, a Black man killed by police in Minneapolis, Minnesota, in May 2020. Protests and traffic closures forced many resources to temporarily close^25^ and potentially altered civilian-police relationships. Previous research has shown that witnessing police brutality decreases the likelihood of reporting crimes by reinforcing the belief that police may in fact escalate violence.^26^ In today’s digital world, widely circulating images of police violence against Black persons may have reduced trust in police, especially in Chicago, where Black persons account for 74% of fatalities due to police violence but comprise only 29% of the city’s population.^27^ Furthermore, many DV survivors are women,^4^ and cases of police violence against Black women specifically, may have contributed to an erosion of trust during the pandemic. For example, news of Anjanette Young, a Black woman in Chicago who encountered police misconduct during an erroneous home raid, was widely reported in the media in 2019 and 2020.^28^

Third, resource availability for mental health and for personal safety decreased during the pandemic, especially in the Black majority south side of Chicago. Domestic violence resources offer considerable support to individuals by empowering DV survivors, helping survivors navigate legal systems, and offering financial, health, and housing support.^29,30^ Therefore, resource reductions may have made it significantly more difficult to prevent, report, or escape DV (eg, accessing safe alternative housing after police intervention). Media reports also found associations between SH measures and increased DV resource demand,^6^ possibly indicating a higher incidence in DV, reduced access to informal resources (eg, staying at a friend’s home), or both. Limited access to mental health resources may also contribute to a rise in DV by limiting support for individuals at high risk of perpetration under stress. Although it is plausible that pandemic-related stress initially unified households against a “common enemy,” prior research has shown that even in the face of exogenous threats, abusive partners often continue to display abusive behavior, whereas those experiencing violence become less likely to report it.^31^

Taking these findings together, we theorize that as civilians were forced to prioritize either health or safety during the pandemic, thresholds for reporting to police changed. Individuals may have avoided reporting out of fear of escalation or retaliation from perpetrators with whom they were now isolated. If police were called but perpetrators were not removed from the residence, individuals may have had nowhere safe to go—family and friends were likely social distancing and shelters may have been perceived as unacceptable risks for acquiring COVID-19. Individuals who were able to rely on other people for safe, alternative housing may have remained at risk for COVID-19 and possibly DV owing to overcrowded housing.^32^ It is also plausible that individuals avoided reporting DV out of fear of acquiring the virus from police officers directly, compounded by media reports of some Chicago officers refusing to wear masks.^33^

### Limitations

There are several limitations to this study. First, we were unable to compare DV reporting rates with incidence rates. Domestic violence incidence is notoriously challenging to estimate given the constraints of self-reporting and the reticence of those experiencing DV to come forward. Second, resource data provided information on availability but not on use or capacity, which was likely heterogeneous across time and communities. In fact, resources may have been “open,” but with reduced capacities to prevent COVID-19 transmission,^34^ thereby reducing use. Third, despite frequent resource database updates, resource availability fluctuated throughout the pandemic and may not be fully reflected in this analysis. Fourth, we were unable to test for mediating effects of DV resource availability on police reporting, owing to differences in the statistical models required for each analysis.

Future research should explore how people experiencing DV navigate community resources as alternatives or adjuncts to police engagement. Our study raises the possibility that reticence to involve police may increase as community resource availability diminishes. Individuals may wish to have long-term strategies in place before engaging police. Currently, Boulder County Public Health in Colorado state is implementing a behavioral health coresponder program to provide immediate behavioral health assessment and connection to resources at the point of contact with police.^35^ Those types of integrated strategies may support longer-term assistance and support of individuals experiencing DV. However, our findings are also consistent with the possibility that police violence and systemic racism increased the reticence of those experiencing DV to report incidents. It is therefore important to explore and support resources that operate independently of police. In addition, comprehensive care for people experiencing DV must attend to the role of structural racism and racial inequities in perpetuating violence.

## Conclusions

This study found that the rate of DV police reporting in Chicago decreased after the March 2020 COVID-19 SH order was issued relative to the same months in 2019. The decreased rate of DV police reporting was substantially greater in majority Black vs majority White communities. Domestic violence resource availability also decreased at higher rates on the city’s majority Black south side. Notably, mental health and personal safety resources constituted the majority of closures due to COVID-19. As the United States overcomes the pandemic, strategies to curb the effects of COVID-19 should include efforts to support individuals affected by DV. Domestic violence resources should be prioritized and supported to maintain services, especially in communities experiencing a high burden of COVID-19 and racial inequity.

## Notes

Supplement. **eTable 1.** Citywide Domestic Crime Reporting Counts and Rates by Time Period, Chicago, IL **eFigure.** Citywide Domestic Violence Reporting Trends by Month Before and After Implementation of Chicago’s Stay-at-Home (SH) Order, Chicago, IL **eTable 2.** Race/Ethnicity Stratified Change in Domestic Violence (DV) Reporting Rates Associated with the COVID-19 Stay-at-Home Order, Chicago, IL **eTable 3.** Citywide Homicide Reporting Counts and Rates by Time Period, Chicago, IL **eTable 4.** Change in Homicide Reporting Counts Associated with the COVID-19 Stay-at-Home Order, Chicago, IL **eTable 5.** Domestic Violence Resource Availability by Resource Type During the COVID-19 Pandemic, Chicago, IL **eTable 6.** Change in Availability of Domestic Violence Resources Associated With the COVID-19 Pandemic, Chicago, IL
