# Supplementary material for: Domestic Violence Police Reporting and Resources During the 2020 COVID-19 Stay-at-Home Order in Chicago, Illinois
Source: JAMA Netw Open. 2021 Sep 2;4(9):e2122260. doi: 10.1001/jamanetworkopen.2021.22260 (PMC8414190; doi:10.1001/jamanetworkopen.2021.22260)
Supplement: Supplement. — eTable 1. Citywide Domestic Crime Reporting Counts and Rates by Time Period, Chicago, IL eFigure. Citywide Domestic Violence Reporting Trends by Month Before and After Implementation of Chicago’s Stay-at-Home (SH) Order, Chicago, IL eTable 2. Race/Ethnicity Stratified Change in Domestic Violence (DV) Reporting Rates Associated with the COVID-19 Stay-at-Home Order, Chicago, IL eTable 3. Citywide Homicide Reporting Counts and Rates by Time Period, Chicago, IL eTable 4. Change in Homicide Reporting Counts Associated with the COVID-19 Stay-at-Home Order, Chicago, IL eTable 5. Domestic Violence Resource Availability by Resource Type During the COVID-19 Pandemic, Chicago, IL eTable 6. Change in Availability of Domestic Violence Resources Associated With the COVID-19 Pandemic, Chicago, IL [file jamanetwopen-e2122260-s001.pdf]

Supplementary Online Content

Baidoo L, Zakrison TL, Feldmeth G, Lindau ST, Tung EL. Domestic violence police reporting and resources during the 2020 COVID-19 stay-at-home order in Chicago, Illinois. *JAMA Netw Open*. 2021;4(9):e2122260. doi:10.1001/jamanetworkopen.2021.22260

eTable 1. Citywide Domestic Crime Reporting Counts and Rates by Time Period, Chicago, IL

eFigure. Citywide Domestic Violence Reporting Trends by Month Before and After Implementation of Chicago's Stay-at-Home (SH) Order, Chicago, IL

eTable 2. Race/Ethnicity Stratified Change in Domestic Violence (DV) Reporting Rates Associated with the COVID-19 Stay-at-Home Order, Chicago, IL

eTable 3. Citywide Homicide Reporting Counts and Rates by Time Period, Chicago, IL

eTable 4. Change in Homicide Reporting Counts Associated with the COVID-19 Stay-at-Home Order, Chicago, IL

eTable 5. Domestic Violence Resource Availability by Resource Type During the COVID-19 Pandemic, Chicago, IL

eTable 6. Change in Availability of Domestic Violence Resources Associated With the COVID-19 Pandemic, Chicago, IL

This supplementary material has been provided by the authors to give readers additional information about their work.

eTable 1. Citywide Domestic Crime Reporting Counts and Rates by Time Period, Chicago, IL

Domestic Crime Type	2019		2020	
	Jan-Mar[a]	Apr-June[a]	Jan-Mar (Pre-SH)[a]	Apr-June (Post-SH)[a]
	No. Crime Reports (per 100,000 ppl)[b]	No. Crime Reports (per 100,000 ppl)[b]	No. Crime Reports (per 100,000 ppl)[b]	No. Crime Reports (per 100,000 ppl)[b]
Violent	7,178 (264.0)	8,515 (313.2)	7,062 (259.8)	6,877 (253.0)
Property	1,260 (46.3)	1,610 (59.2)	1,358 (50.0)	1,595 (58.7)
Other	1,435 (52.8)	1,440 (53.0)	1,123 (41.3)	1,005 (37.0)
Total	9,873 (363.2)	11,565 (425.4)	9,543 (351.0)	9,477 (348.6)
Pearson chi2(6) = 125.4; p<0.001				

[a]'Pre-SH' indicates the 3-month time period before the Stay-at-Home (SH) order was implemented (Jan-Mar), while 'Post-SH' indicates the 3-month time period after the SH order was implemented (Apr-Jun). [b]Rates per 100,000 people calculated using an estimated population of 2,718,555 for the 77 Chicago community areas reflected in the July, 2020 release of Chicago Metropolitan Agency for Planning (CMAP: https://www.cmap.illinois.gov) data.

© 2021 Baidoo L et al. *JAMA Network Open*.

eFigure. Citywide Domestic Violence Reporting Trends by Month Before and After Implementation of Chicago's Stay-at-Home (SH) Order, Chicago, IL

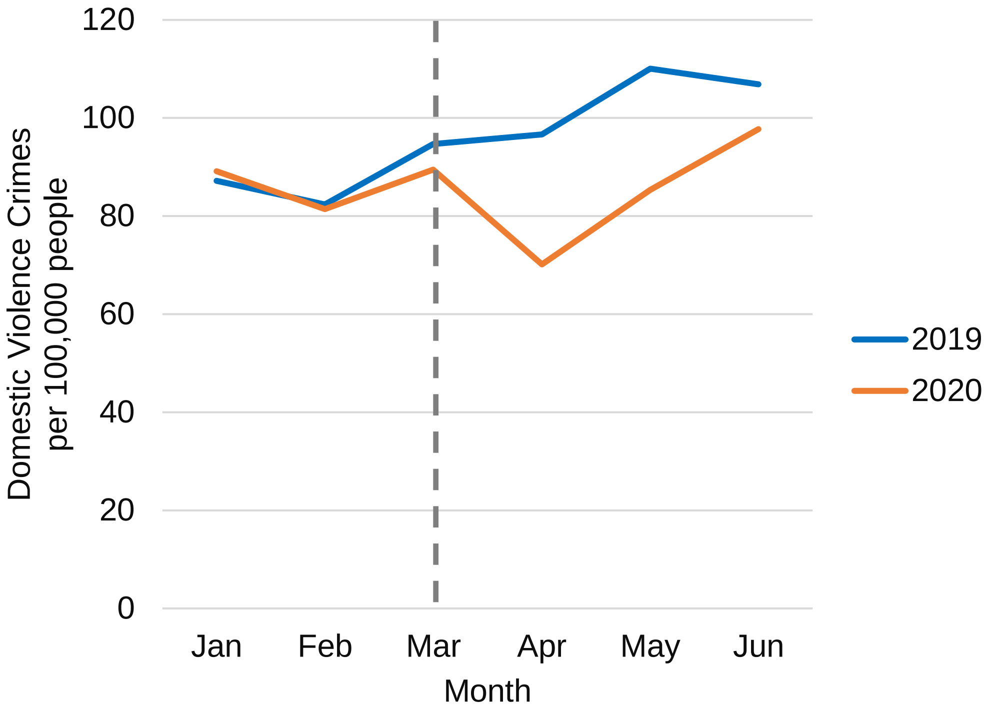

Fig. shows the trend in rates of police reporting for domestic violence by month during the study period, comparing 2020 (orange) to the same time period in 2019 (blue), before and after implementation of Chicago's SH order (vertical dash line).

© 2021 Baidoo L et al. *JAMA Network Open*.

eTable 2. Race/Ethnicity Stratified[a] Change in Domestic Violence (DV) Reporting Rates Associated with the COVID-19 Stay-at-Home Order, Chicago, IL

**Crime Type**	**Racial/Ethnic Majority Composition**	Change in DV reports per 100,000 ppl[b]	[95% CI]	P-value
Domestic Violence	White	-4.4	[-21.64, 12.83]	0.62
	Black/African-American	-45.2	[-59.06, -31.43]	<0.001
	Hispanic/Latinx	-8.2	[-25.02, 8.52]	0.34
	Other/None[c]	-14.5	[-35.59, 6.62]	0.18

[a]A non-factorial interaction term was implemented (in addition to models using factorial design presented in Table 2), to test for any significant changes in DV reporting within White majority community areas. [b]Model implemented mixed effects linear regression models to calculate the average change in police reporting rates (per community area per month) as a function of the interaction between time period (pre-SH vs. post-SH) and year (2019 vs. 2020), controlling for median age, median household income, educational attainment, vehicle access, and group transportation; reporting rates were additionally stratified (non-factorial) by each community area's racial/ethnic composition. [c]"Other" includes community areas with a majority of residents identifying as Non-Hispanic Asian, Native Hawaiian and Other Pacific Islander, American Indian and Alaska Native, "some other race," or multiracial, as designated by the U.S. Census Bureau's American Community Survey.

© 2021 Baidoo L et al. *JAMA Network Open*.

**eTable 3.** Citywide Homicide Reporting Counts and Rates by Time Period, Chicago, IL				
	**2019**		**2020**	
**Crime Type**	**Pre-SH**[a]	**Post-SH**[a]	**Pre-SH**[a]	**Post-SH**[a]
	No. Crime Reports (per 100,000 ppl)[b]	No. Crime Reports (per 100,000 ppl)[b]	No. Crime Reports (per 100,000 ppl)[b]	No. Crime Reports (per 100,000 ppl)[b]
Domestic Homicide	7 (0.26)	8 (0.29)	8 (0.29)	13 (0.48)
Non-Domestic Homicide	75 (2.76)	156 (5.74)	91 (3.35)	224 (8.24)
Total Homicide	82 (3.02)	164 (6.03)	99 (3.64)	237 (8.72)
Pearson chi2(3) = 2.0073; p-value = 0.557				

[a]'Pre-SH' indicates the 3-month time period before the Stay-at-Home (SH) order was implemented (Jan-Mar), while 'Post-SH' indicates the 3-month time period after the SH order was implemented (Apr-Jun). [b]Rates per 100,000 people calculated using an estimated population of 2,718,555 for the 77 Chicago community areas reflected in the July, 2020 release of Chicago Metropolitan Agency for Planning (CMAP: https://www.cmap.illinois.gov) data.

© 2021 Baidoo L et al. *JAMA Network Open*.

eTable 4. Change in Homicide Reporting Counts Associated with the COVID-19 Stay-at-Home Order, Chicago, IL

Homicide Type	Racial/Ethnic Majority Composition	Model 1[a]			Model 2[b]		
		Change in Homicide	[95% CI]	P-value	Change in Homicide	[95% CI]	P-value
Domestic Homicide		0.0	[-0.04,0.07]	0.53			
	White				Ref		
	Black/African-American				0.05	[-0.09,0.19]	0.51
	Hispanic/Latinx				0.00	[-0.15,0.15]	1.00
	Other/None[c]				0.00	[-0.17,0.17]	1.00
Total Homicide		0.2	[0.01,0.48]	0.04			
	White				Ref		
	Black/African-American				0.52	[-0.09,1.12]	0.09
	Hispanic/Latinx				0.23	[-0.43,0.89]	0.50
	Other/None[c]				0.10	[-0.65,0.85]	0.79

[a]Model 1 implemented mixed effects linear regression models to calculate the average change in homicide reporting counts (per community area per month) as a function of the interaction between time period (pre-SH vs. post-SH) and year (2019 vs. 2020), controlling for median age, median household income, educational attainment, vehicle access, and group transportation. [b]Model 2 implemented all conditions of Model 1 but additionally stratified by each community area's racial/ethnic composition. [c]"Other" includes community areas with a majority of residents identifying as Non-Hispanic Asian, Native Hawaiian and Other Pacific Islander, American Indian and Alaska Native, "some other race," or multiracial, as designated by the U.S. Census Bureau's American Community Survey.

© 2021 Baidoo L et al. *JAMA Network Open*.

**eTable 5.** Domestic Violence Resource Availability by Resource Type During the COVID-19 Pandemic, Chicago, IL						
**Service Type** No. (%)	**Phone/ Virtual**[a]	**Regular Operations**[a]	**Regular Operations & Phone/ Virtual**[a]	**Service Unavailable**	**Unable to Verify**	**Total**
**Legal Assistance**						
Advocacy and court accompaniment	8 (80.0)	1 (10.0)	0 (0.0)	0 (0.0)	1 (10.0)	10
**Mental Health**						
Counseling services	216 (53.9)	23 (5.7)	78 (19.5)	18 (4.5)	66 (16.5)	401
**Personal Safety**						
Anger management classes	21 (46.7)	7 (15.6)	3 (6.7)	6 (13.3)	8 (17.8)	45
DV prevention education	23 (65.7)	4 (11.4)	1 (2.9)	5 (14.3)	2 (5.7)	35
Domestic violence shelter	0 (0.0)	3 (100.0)	0 (0.0)	0 (0.0)	0 (0.0)	3
Survivor support services	14 (70.0)	0 (0.0)	4 (20.0)	2 (10.0)	0 (0.0)	20
**Hotlines**						
Hotline - Crisis help[a]	2 (100.0)	-	-	0 (0.0)	0 (0.0)	28
Hotline - Domestic	6 (100.0)	-	-	0 (0.0)	0 (0.0)	6
Hotline - Sexual assault[a]	4 (100.0)	-	-	0 (0.0)	0 (0.0)	4
**Total**	320 (58.0)	38 (6.9)	86 (15.6)	31 (5.6)	77 (13.9)	552

[a]Phone/Virtual, Regular Operations, and Regular Operations & Phone/Virtual considered to be equivalent COVID-19 statuses.

© 2021 Baidoo L et al. *JAMA Network Open*.

eTable 6. Change in Availability of Domestic Violence Resources Associated With the COVID-19 Pandemic, Chicago, IL

**Geographic Area**	Change in Resources per 100,000 ppl[a]	[95% CI]	P-value
North Side	Ref	-	-
South Side	-6.7	[-12.92, - 0.46]	0.04
West Side	-3.6	[-10.35, 3.15]	0.30

[a]Model implemented mixed effects linear regression models to calculate the average change in resource availability (per community area) as a function of time period (pre-COVID vs. COVID), controlling for median age, median household income, educational attainment, vehicle access, and group transportation; resource availability was additionally stratified by each community area's geographic region (North, South, or West Side of Chicago).

© 2021 Baidoo L et al. *JAMA Network Open*.
